# What the X Has to Do with It: Differences in Regulatory Variability between the Sexes in *Drosophila simulans*

**DOI:** 10.1093/gbe/evu060

**Published:** 2014-04-01

**Authors:** Rita M. Graze, Lauren M. McIntyre, Alison M. Morse, Bret M. Boyd, Sergey V. Nuzhdin, Marta L. Wayne

**Affiliations:** ^1^Department of Molecular Genetics and Microbiology, University of Florida; ^2^Department of Biological Sciences, Auburn University; ^3^Department of Statistics, University of Florida; ^4^Florida Museum of Natural History, University of Florida; ^5^Section of Molecular and Computational Biology, Department of Biological Sciences, University of Southern California; ^6^Department of Biology, University of Florida

**Keywords:** *Cis*/*trans* gene regulation, allele-specific expression, sex-biased expression, X-chromosome

## Abstract

The mechanistic basis of regulatory variation and the prevailing evolutionary forces shaping that variation are known to differ between sexes and between chromosomes. Regulatory variation of gene expression can be due to functional changes within a gene itself (*cis*) or in other genes elsewhere in the genome (*trans*). The evolutionary properties of *cis* mutations are expected to differ from mutations affecting gene expression in *trans*. We analyze allele-specific expression across a set of X substitution lines in intact adult *Drosophila simulans* to evaluate whether regulatory variation differs for *cis* and *trans*, for males and females, and for X-linked and autosomal genes. Regulatory variation is common (56% of genes), and patterns of variation within *D. simulans* are consistent with previous observations in Drosophila that there is more *cis* than *trans* variation within species (47% vs. 25%, respectively). The relationship between sex-bias and sex-limited variation is remarkably consistent across sexes. However, there are differences between *cis* and *trans* effects: *cis* variants show evidence of purifying selection in the sex toward which expression is biased, while *trans* variants do not. For female-biased genes, the X is depleted for *trans* variation in a manner consistent with a female-dominated selection regime on the X. Surprisingly, there is no evidence for depletion of *trans* variation for male-biased genes on X. This is evidence for regulatory feminization of the X, *trans*-acting factors controlling male-biased genes are more likely to be found on the autosomes than those controlling female-biased genes.

## Introduction

There are greater contributions of *cis* than *trans* variants to interspecific divergence in expression regulation (Wittkopp et al. 2004; Lemos et al. 2008; Graze et al. 2009; Tirosh et al. 2009; Emerson et al. 2010; McManus et al. 2010). However, the story within species is less clear: although there is abundant regulatory variation (Townsend et al. 2003; Morley et al. 2004; Wayne et al. 2004), there are contradictory findings on the relative importance of *cis* versus *trans* variation (Brem et al. 2002; Schadt et al. 2003; Hughes et al. 2006; Genissel et al. 2008; Lemos et al. 2008; Wang et al. 2008; Wittkopp et al. 2008b). The lack of consensus may reflect differences between experimental designs: studies that use expression QTL (eQTL) designs or multiple chromosome substitutions have found many more *trans*-acting variants than *cis*-acting variants (Brem et al. 2002; Schadt et al. 2003; Genissel et al. 2008; Wang et al. 2008). Other approaches (primarily single chromosome substitutions and allele-specific expression [ASE] studies) have found much more evidence of *cis*-regulatory variation than *trans*-acting variation (Lemos et al. 2008; Wittkopp et al. 2008b).

Regulatory variation arising on the X chromosome will be affected by the unique evolutionary properties of the X (for review see Vicoso and Charlesworth 2006), in addition to differences between *cis* and *trans* mutations. In Drosophila, hemizygosity of the X chromosome in males results in different evolutionary trajectories for X-linked genes relative to autosomal genes due to differences in population size, average recombination rate, and dominance variation (Hedrick and Parker 1997; Begun et al. 2007; Mackay et al. 2012). The selective regime of X-linked genes also differs from that of autosomal genes: they spend more time in females than in males. Also, the X chromosome is subject to hemizygosity in males, which should increase the efficiency of selection for X-linked genes (Begun and Whitley 2000; Baines et al. 2008; Singh et al. 2008) as long as there is at least partial dominance. In addition, partially recessive (or dominant) mutations with sexually antagonistic effects (i.e., alleles that are beneficial in one sex but deleterious in the other[Rice 1984; Chippindale et al. 2001; Gibson et al. 2002]) are expected to experience decreased time to both fixation and extinction on the X (but see Fry 2010). Indeed, a recent study directly linking transcript abundance with sex-specific fitness suggests that the X chromosome is enriched for sexually antagonistic genes (Innocenti and Morrow 2010), but such genes still make up a very small percentage of the genome (perhaps 8%).

It is perhaps surprising then that studies of gene expression conclude that the X chromosome, far from being enriched for male-benefiting alleles (i.e., masculinized), is both depauperate for male-biased genes and appears to be enriched for female-benefiting alleles (i.e., feminized [Parisi et al. 2003; Ranz et al. 2003]). Implicit in these interpretations was the assumption that sex-biased expression (i.e., expression that is greater in one sex than the other) translates into differential function between the two sexes (e.g., the sex with higher expression is the sex whose fitness is affected most by the transcript). Phenotypic data now explicitly relate sex-biased expression to sex-specific fitness, at least in the case of mutations of large effect (visible, sterile, and lethal; Connallon and Clark 2011). Genes with fitness effects that are either limited to or are larger in females tend to have female-biased expression. Similarly, genes with fitness effects limited to or more extreme in males tend to be male-biased genes. However, genes with effects that are similar between the sexes also tend to be female biased. Thus, female bias is not a priori evidence for sexual antagonism, and moreover, suggests that the feminization of the X may have nothing to do with sexual antagonism.

By dissecting standing variation for gene expression using a classical X substitution design, we provide insights into how regulatory variation is shaped by sex and X chromosome evolution. We examine expression across the whole genome in both sexes for *cis-* and *trans*-regulatory variation within *Drosophila simulans*. We conclude that chromosomal context shapes *cis* and *trans* variation, depleting *cis* and *trans* variation among X-linked genes relative to the autosomes, consistent with stronger purifying selection on the X than the autosomes. *Cis* and *trans* variation are also frequently sex specific, and this is related to sex-biased gene expression. Purifying selection appears to erode *cis* variation within the sex toward which expression is biased (i.e., among male-biased genes, there is greater female-specific *cis* variation than male-specific variation, and vice versa). Interestingly, there is more female-specific variability for both *cis* and *trans* variation, implying that standing regulatory variation differs fundamentally between the sexes.

## Materials and Methods

### X-Substitution Line Construction

A common isogenic reference background (*st e*) was created from a stock *st e* line, DSSC 14021-0251.041, by single pair full-sib mating for more than 20 generations (Graze et al. 2007). The X chromosomes of five *D. simulans* parental lines (P) sequenced by the Drosophila Population Genomics Project (Begun et al. 2007; *w*501, DSSC 14021-0251.195; NewC, DSSC 14021-0251.198; MD199S, DSSC 14021-0251.197; MD106ts, DSSC 14021-0251.196; C167.4, DSSC 14021-0251.199) were introgressed into the common *st e* genetic background, creating five X-substitution lines. A total of 68 substitution lines (X^sub^ lines) were created initially, and homozygosity was assayed by restriction fragment length polymorphism (RFLP) and by sequencing at two loci on either end of the substituted X (CG1636 and CG32599). Only lines homozygous for the substitution were used in the experimental crosses.

### Experimental Design and Sample Collection

**Fig. 1.—:**
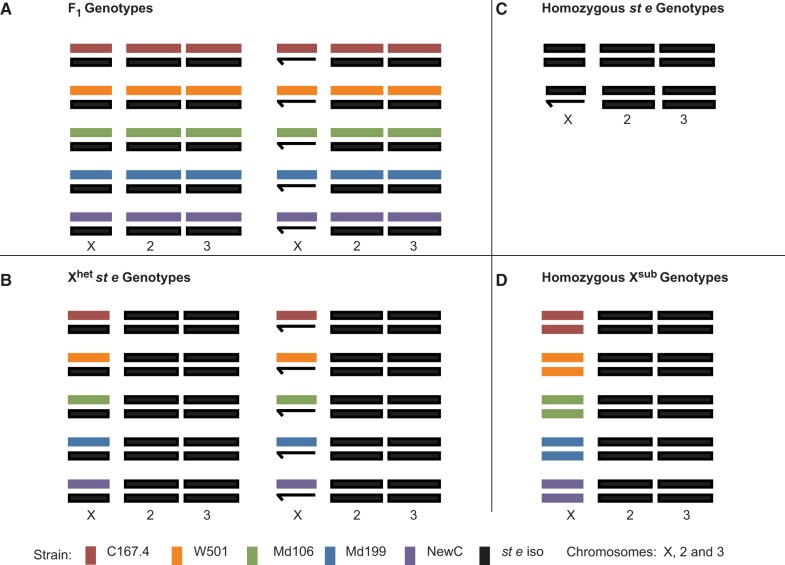
Experimental design. Genotypes used in the experiment were produced from 5 parental *D. simulans* strains (C167.4, Md106, Md199, NewC, and w501), 5 corresponding X-substitution lines (denoted X^sub^), and 1 reference strain (*st e*). For each parental *D. simulans* strain, X chromosomes were substituted into the common isogenic (*st e*) background. Each of the parental strains was crossed to the *st e* line to produce five F_1_ genotypes (X^sub^X^st e^ A^sub^A^st e^, X^sub^Y^st e^ A^sub^A^st e^) that were heterozygous (or hemizygous) for both X and the autosomes (panel *A*). Each of the X-substitution lines was crossed to the *st e* line creating five X^het^ *st e* genotypes (X^sub^X^st e^ A^st e^A^st e^, X^sub^Y^st e^ A^st e^A^st e^) that were heterozygous (or hemizygous) for X only (denoted X^het^ *st e*; panel *B*). Six genotypes (X^sub^X^sub^ A^st e^A^st e^, X^st e^X^st e^ A^st e^A^st e^, X^st e^Y^st e^ A^st e^A^st e^) that were homozygous for both X and autosome (panels *C* and *D*) were also included in the experiment.

Flies were reared in incubators (25 °C, 12:12 h light/dark cycle) on a standard dextrose medium at standardized densities for at least two generations. Stocks of the five parental lines, the *st e* common reference line, and the five X-sub lines were crossed to produce the genotypes used in the experiment (fig. 1). For each cross, 20 virgin females were crossed to five males. For crosses involving the X^sub^ or P lines and the *st e* line, the female parent was always X^sub^ so that all male progeny, who are hemizygous for the X, contained the substituted X rather than X*^st^^e^*. Three cross types were used in this study: homozygotes, which are the homozygous progeny of the five X^sub^ × X^sub^ crosses and of the reference line (*st e* × *st e*); F_1_, which are the progeny of each of the five original P lines to *st e,* producing progeny heterozygous for both X and the autosomes; and X^het^ *st e*, which are the progeny of the cross of each of the five X^sub^ lines to *st e* homozygotes, producing progeny heterozygous for the X chromosome only in an otherwise homozygous *st e* (reference) background. Upon eclosion, flies were sexed and separated into separate vials using CO_2_ anesthesia and aged 5–7.5 days. Collections occurred in a single 2.5-h window from 4:00 to 6:30 PM. The total number of crosses was small enough that collections for all crosses were conducted simultaneously.

For RNA samples, two sets of 20 flies (subsamples) were collected for each replicate from multiple rearing vials. For each genotype and sex, three independent replicate RNA samples were constructed. Additional data, generated concurrently with all other samples described, were included in the final analysis (six RNA samples hybridized, three replicates each for females and for males, of the F_1_ of C167.4 and *st e* only; Yang et al. 2011). A total of 81 RNA samples were hybridized: 3 replicates × 2 sexes × 11 genotypes (5 F_1_ genotypes, 5 X^het^ *st e* genotypes, and the homozygous *st e* genotype; total of 66 samples); and three replicates for females only for five homozygous X^sub^ genotypes (15 samples), as male homozygotes are genetically identical to males from X^het^ *st e*.

A single DNA control sample from a minimum of 40 females was made for each homozygous genotype and each F_1_ genotype, for a total of 11 DNA samples for hybridization (fig. 1). Additionally, three DNA samples from the F_1_ of C167.4 and *st e* only, as well as three DNA samples from *st e* homozygotes, were generated concurrently with the other samples described and were included in the analysis (Yang et al. 2011).

### Sample Processing

All RNA extractions and quality assessments were carried out as described in Yang et al. (2011). Genomic DNA was isolated from flash-frozen flies using Qiagen’s DNeasy Blood and Tissue Kit following the manufacturer’s protocol, treated with RNase (4 μl of 100 mg/ml RNase A, 2 min r.t. incubation), and purified by phenol/chloroform extraction. Fragmentation, labeling, and array hybridization for RNA and for DNA were carried out as in Yang et al. (2011), with the following modification; for each DNA sample, 10 μg of DNA was fragmented, and 9 μg was labeled and hybridized. To measure expression, exon level signal, and variation in ASE, a custom microarray platform was constructed containing three different modules: 3′ expression probe sets (*n* = 18,769 Perfect Match (PM), probe sets from the Affymetrix Drosophila Genome 2.0 Array design); exon probe sets (*n* = 61,919 probe sets corresponding to exonic regions from the Affymetrix Drosophila Tiling 2.0 Array design); and custom single nucleotide polymorphism (SNP) probe sets (*n* = 61,752) for *D. simulans* SNPs; the array also contained the standard Affymetrix hybridization control probes and the GC bin controls (Yang et al. 2011). Signal for each probe in each of the three modules was extracted (Yang et al. 2011). Quality control (QC) was conducted as described in Yang et al. (2011), and no problems with hybridization quality were identified. Probe sequences and chip annotation can be found at gene expression omnibus (GEO) using accession ID GPL11273. The GEO accession for the array data is GSE31750. After overall quality control, probes in the 3′ expression set, the exon set, and the SNP set were separated for analysis.

### Analysis

#### Analysis of Overall Expression

To assay differences in transcript abundance levels between genotypes and between sexes, total transcript level was assayed using the 3′ expression module (Affymetrix Drosophila 2.0 PM probe sets). A total of 18,769 probe sets were analyzed, allowing transcript level to be assayed for 12,931 FlyBase R5.11 annotated genes. For each probe in a probe set, the GC content was used to identify the corresponding mismatch (MM) control probes. The fifth percentile of the MM probes was subtracted from each perfect match (PM) probe and the average intensity value for the probe set calculated. The natural log of the mean +100 was used as the estimate of expression.

For each probe set, a cell means model, *Y_ij_* = µ + *t_i_* + *ε_ij_* , was fit, where the dependent variable Y*_ij_* is the normalized expression for each of the *i* genotypes and *j* replicates for RNA hybridizations only. Males and females from the same cross are considered separate genotypes. Individual contrasts were constructed to test the null hypothesis that the homozygous X^sub^ genotype had the same expression as in *st e*. Overall expression (OE) in chromosome substitution lines can be used to infer *cis* and *trans* effects (e.g., Lemos et al. 2008). For homozygous X-substitution line comparisons of differences in OE, the contrasts test *cis* effects for genes on X (the substituted chromosome) and *trans* effects for autosomal genes. Contrasts evaluating dominance were constructed as tests of the heterozygote versus the expected midparent mean (for X^sub^ parents and progeny only). Contrasts were also constructed for 1) an overall test of the effect of X variability among the genotypes and 2) an overall test for sex effects. Genes were classified as sex biased if the null hypothesis that average expression of males was equal to the average expression of females was rejected. Sex-biased genes were further classified as female/male biased based upon the estimated difference in the means. The false discovery rate (FDR) for all tests in the 3′ IVT expression set was determined by simultaneously considering all contrasts (Benjamini and Hochberg 1995); for review, see Verhoeven et al. (2005). To balance false negatives and false positives, an FDR of 0.20 was considered significant. Other levels were considered and overall trends are unaffected by this choice. Raw *P* values and FDR-adjusted *P* values are given in supplementary file S1, Supplementary Material online.

#### Analysis of ASE

When expression of the two alleles in a heterozygote is significantly different (termed allelic imbalance or AI), *cis* differences between alleles can be inferred since the *trans* environment is the same for both alleles. Examining the same allele in two cellular environments can reveal *trans* variation. Interactions are not separable from main effects in these designs (Wang et al. 2008; Wittkopp et al. 2008a; Graze et al. 2009). The contribution of *cis* by *trans* interactions can be identified by comparing composite *cis* effects between genotypes with different *trans* backgrounds (Wittkopp et al. 2008a). To account for technological limitations, DNA controls have been used with pyrosequencing (Wittkopp et al. 2004), tiling arrays (Graze et al. 2009), and RNA seq (Graze et al. 2012). We hybridized DNA samples as controls in this experiment (see supplementary fig. S1, Supplementary Material online).

In order to estimate ASE, expression must be measured individually for each allele. ASE was estimated from SNP probe set signals in RNA hybridizations (Yang et al. 2011). For genotypes X^sub^X^st e^A^sub^A^st e^, X^sub^Y^st e^A^sub^A^st e^ (F_1_), and X^sub^X^st e^A^st e^A^st e^ (X^het^ *st e*), the chromosomes are derived from different parental lines. There were a total of 61,752 SNP probe sets on the array developed from population genomic data (DPGP, http://www.dpgp.org, last accessed April 2, 2014; Begun *et al.* 2007) with 24 probes in each SNP probe set, all four bases, forward and reverse strands are represented for three positions in the probe set (0, +4, −4) (Yang et. al. 2011; Affymetrix array 520726). The SNP alleles were assigned to perfect match 1 (PM1), perfect match 2 (PM2), and MM probes. For each cross and probe set combination, if the resequencing data (Begun et al. 2007) showed an SNP between the two parents, the PM1 and PM2 probes were assigned to the matching parental alleles (*st e* or allele2). If there was no polymorphism, the probe set was not analyzed further for that cross. If the *st e* allele was available but did not match either SNP allele, the probe set was not analyzed further. If one or both of the parents was missing resequencing data, linear discriminant analysis (LDA) was used to infer whether the cross was polymorphic. LDA is a multivariate technique that uses distance separation to classify continuous observations into categorical groups. We applied LDA, assuming that the RNA from the two parental genotypes represented different SNP bases. If the LDA successfully identified the F_1_ as a heterozygote or both parents were unambiguously identified as different homozygotes, the probe set was retained and the PM1 and PM2 probes assigned to ste/allele2. Otherwise, the probe set was not analyzed further for that cross. For each probe set, the average signal for the ste/allele2/MM probes was calculated and normalized by taking the natural log of the signal value and subtracting the median value from the SNP probe sets for that slide.

**Fig. 2.—:**
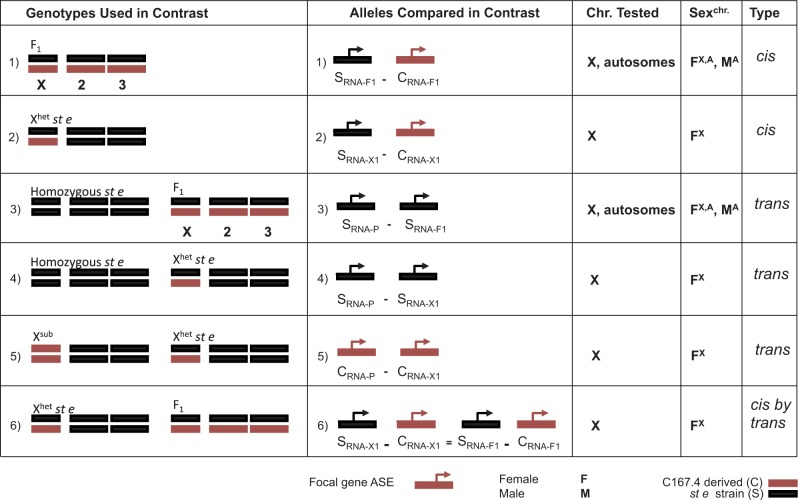
Allele-specific analysis of *cis* and *trans* variation. For each contrast 1–6: the genotypes (only c167.4 shown) used in a contrast are shown in the first column; for a given focal gene, the allele-specific expression measurements used in the test are given in the second column, noted as C (allele derived from the C167.4 parental strain) or S (allele derived from the *st e* reference strain) in the genotype indicated by subscripts P (parental strain), F1 (F_1_), or X1 (X^het^ *st e*); the genes that could be tested, X-linked (X) and autosomal (Autosome), are listed in the third column; the sex, Male (M) and Female (F), that the test could be conducted for is listed in the fourth column (with the genes that could be tested in superscript); and the effect tested is listed in the fifth column. Note that the *cis* by *trans* test was considered significant only if the *cis* effect in contrasts 1 or 2 was nonzero. For each *cis* or *trans* test, the difference in expression between the two alleles was compared with the allele-specific signal in the appropriate DNA control (supplementary fig. S1, Supplementary Material online).

For a single gene, all the probe sets that separated the alleles (*st e* and X^sub^) for that gene were considered jointly and tested for *cis* and *trans* effects. In autosomal genes, a cell means model, *Y_ijklm_* = µ + *t_ijkl_* + *ε_ijklm_*, was fit. The dependent variable Y*_ijklm_* is the normalized allele-specific signal for each of the *i* alleles (*st e* or X^sub^), *j* genotypes (1-C167.4, 2-MD106ts, 3-MD199S, 4-NewC, 5-*w*501), *k* nucleic acids (DNA or RNA), and *l* sexes (male or female) for *m* replicates (1, 2, 3). To account for heteroscedasticity, separate variances for DNA and RNA were specified. For genes on the X, the model is the same, except that the *l* term sex is not included, as only females can be tested for AI for genes on the X. *F*-tests for *cis* and *trans* effects were constructed as contrasts from this cell means model (fig. 2). The *F*-test of *cis* effects for autosomal genes in c167.4 F_1_ females (fig. 2, row 1) tests the difference between the *st e* allele and the c167.4 allele relative to the difference observed in the DNA control (following Graze et al. 2009; Wittkopp et al. 2004). For this test, the null hypothesis is: μ_ste,1,R,F_ – μ _c167.4,1,R,F = _μ _ste,1,D,F_ – μ _c167.4,1,D,F_. Similarly, contrasts were constructed for each of the tests listed in figure 2. All of the contrasts were estimated from one model for autosomes and one model for the X. Tests were grouped by contrast type (fig. 2) and corrected for multiple testing using an FDR (Benjamini and Hochberg 1995). We used an FDR of 0.20 to balance type I and type II error probabilities and to allow for more powerful testing of association; however, other levels (0.10, 0.05) were also examined, and the results were qualitatively similar. All results including raw *P* values, FDR corrected *P* values, estimates of effect size, and determination of significance are provided in supplementary file S2, Supplementary Material online. Full details of the analysis and all analytical programs may be found at: http://bioinformatics.ufl.edu/McIntyre_Lab_7/node/839 (last accessed April 2, 2014)*.* Finally, there is no mean–variance relationship for expression level, nor is there any other evidence that expression level is confounded with detection of *cis*, *trans*, and *cis* × *trans* interactions.

## Results

### More Variation in Transcript Abundance Is Contributed by *Cis*-Regulatory Variants than by *Trans*-Acting Factors

Regulatory variation (*cis*, *trans,* or *cis* by *trans*) is present if a gene shows significant *cis* or *trans* effects in any of the five examined *D. simulans* genotypes (c167.4, md106, md199, newc and *w*501). More than half of all genes examined (56%, 6,356 of 11,293) showed evidence of variation in gene regulation within *D. simulans*, which we can attribute to genetic variation in *cis-*regulatory regions, *trans-*acting factors, or *cis* by *trans* genetic interactions (table 1 and fig. 2; supplementary fig. S2*A–C*, Supplementary Material online). This can be considered a minimum estimate of regulatory variation within *D. simulans*, given that it stems from a sample of only six parental genotypes. In addition, *cis*-regulatory variation could be present that is undetectable when comparing OE levels (Pastinen 2010). These results, separated by parental genotype, are summarized in supplementary tables S1–S5, Supplementary Material online.

**Table 1:** *Cis*, *Trans,* and *Cis* by *Trans* Variation in *D. simulans*

Contrast	Sex	Chromosome	Genes Significant	Genes Tested	Percentage Significant
1-*Cis* in F_1_	F	X	501	1,633	30.68
1-*Cis* in F_1_	F	Autosomes	4,103	9,658	42.48
1-*Cis* in F_1_	M	Autosomes	3,704	9,658	38.35
2-*Cis* in X^het^ *st e*	F	X	353	1,633	21.62
3-*Trans* in F_1_	F	X	250	1,633	15.31
3-*Trans* in F_1_	F	Autosomes	1,804	9,660	18.67
3-*Trans* in F_1_	M	Autosomes	1,399	9,660	14.48
4-*Trans* in X^het^ *st e* (st e allele)	F	X	21	1,633	1.29
5-*Trans* in X^het^ *st e* (X-sub allele)	F	X	121	1,633	7.41
6-*Cis* by *trans* interaction	F	X	162	1,633	9.92

Note.—There is a larger percentage of *cis* variation compared to *trans* variation. Results are reported for specific contrasts individually (fig. 2), separated by sex and chromosome.

Although 3,577 of 11,291 (32%) genes tested showed evidence of variation only in *cis* regulation, 1,009 of 11,293 (9%) genes showed evidence of only *trans*-regulatory variation and 1,770 (16%) showed evidence of both. Regardless of whether we examined F_1_ or X^het^ *st e* genotypes (table 1; supplementary fig. S2*A–C*, Supplementary Material online), males or females (table 1; supplementary figure S2*A–C*, Supplementary Material online), or individual X^sub^ parental lines and their progeny (supplementary tables S1–S3, Supplementary Material online), there were always more genes whose expression differed due to *cis*-regulatory variants than *trans*-acting variants. Interestingly, *cis* and *trans* regulation are not independent, and significantly positively covary (Fisher’s exact test, *P* < 0.0001).

*Cis* and *trans* variation may also be evaluated via linkage using a chromosome substitution design. Concurrent with the F1 experiment, we also evaluated X-substitution genotypes for OE. Using the X-substitution approach, variability in expression among genes on the X itself is expected to be largely due to *cis* effects along with some X-linked *trans* effects, whereas variability in expression for genes on the autosomes must be due to *trans*-acting factors on the X. Using OE, *trans* effects on autosomal genes are the result of the combined effect of the *trans*-acting factor on both alleles, while ASE measures the *trans* effects on a single allele.

Here we compare the general pattern of *cis*/*trans* variation that is inferred from the tests of OE with those from ASE. For OE, we inferred *cis and trans* function in X-substitution genotypes from chromosomal location: differences in X-linked genes are expected to largely be *cis*, though we cannot rule out contributions of *trans* variation. Differences in autosomal genes in X-substitution lines, however, are clearly due to *trans* variation. In females only (males only), 38% (22%) of X-linked genes (inferred *cis*) vary in expression; while 30% (21%) of autosomal genes (inferred *trans*) vary in expression. Across males and females, 49% of genes on X (*cis*) varied in expression and 44% of genes on autosomes varied (*trans*). As seen with ASE, for OE, *cis* variation is greater than *trans* variation. However, while the two approaches are qualitatively similar, there are quantitative differences between the approaches. Direct comparisons can be made for *cis* tests for females for X-linked genes (31% ASE, 38% OE). For *trans* tests on the autosomes, we can compare both sexes (females ASE 19%, OE 30%; males 14% ASE, 21% OE). For both *cis* and *trans*, more genes are inferred to vary in regulation in using the OE approach. Hereafter, we will focus on the ASE results.

**Fig. 3.—:**
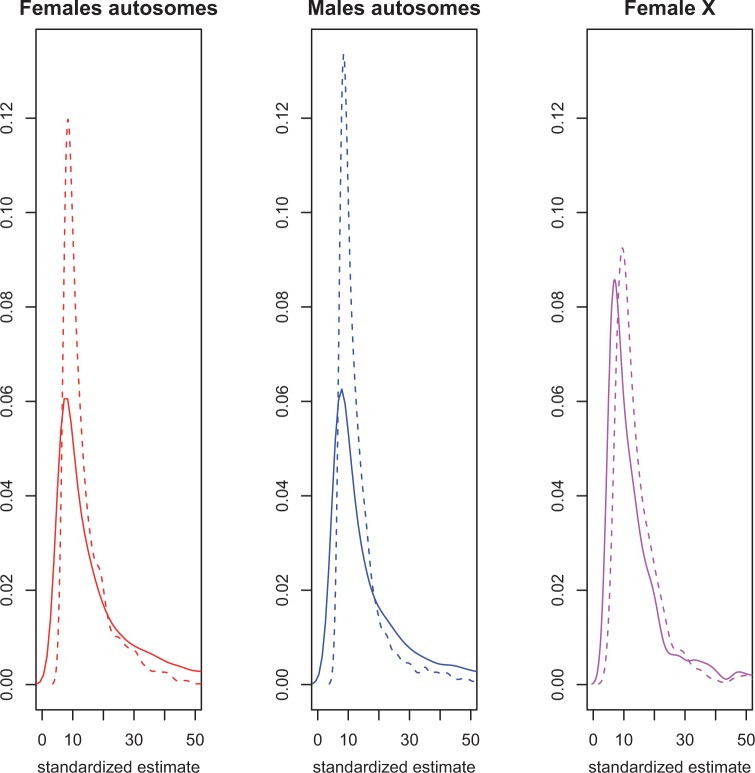
The distribution of *cis* and *trans* variation in transcript abundance. The distribution of the *cis* (solid line) and *trans* (dashed line) effect estimates (calculated as the standardized mean difference) for genes with significant regulatory variation are shown for males (blue) and females (red). The left panel shows the distribution for autosomal genes in females (*n* = 4,103 for *cis* and *n* = 1,804 for *trans*), the middle panel shows the distribution for autosomal genes in males (*n* = 3,704 for *cis* and *n* = 1,399 for *trans*), and the right panel shows the distribution for X-linked genes in females (*n* = 501 for *cis* and *n* = 250 for *trans*). For each plot, the *Y* axis is the frequency and the *X* axis is the standardized estimate of *cis* or *trans* differences between X-substitution parental strain genotypes and the *st e* reference line.

*Cis* and *trans* estimates were more frequently negative than positive, indicating that there were more cases where the *st e* derived allele was expressed at a lower level than the other allele. The mean effect sizes of *cis* and *trans* effects are the same. However, much larger *cis* effects than *trans* effects were detected on the autosomes, and thus the range of effect sizes for *cis* effects is greater. The range of effect sizes for *cis* and *trans* in females on the X is more similar, although there are still twice as many significant *cis* effects as *trans* effects (fig. 3). This is unlikely to be due to a difference in power. If mean *trans* effects are generally smaller than mean *cis* effects (Genissel et al. 2008; Gruber et al. 2012), one would expect to find only large significant *trans* effects, as the power to detect *trans* might be less than for *cis*. Another possibility is that effect sizes are the same, but there is greater error variance for *trans* effects than for *cis* effects. In this case, fewer significant effects of a given size would be detected for *trans* than for *cis* would be detected. We find that the average standardized effect sizes for *cis* and *trans* are the same, indicating that power to detect differences is similar in this design (fig. 3).

Because the *cis*-regulatory regions in X^sub^ homozygotes and in F_1_ heterozygotes are the same in females, we are able to test explicitly for *cis* by *trans* interactions in X-linked genes. Differences in expression between homozygote and heterozygote females must result from interactions between their identical *cis* regions with *trans*-acting variants that differ between the two genotypes (fig. 2, contrast 6). For these genes, 100 of 1,633 (6%) showed evidence of *cis* by *trans* interactions contributing to regulatory variation within *D. simulans*. Again, this estimate is a lower bound of the prevalence of *cis* by *trans* interactions between regulatory variants in *D. simulans*.

### Sex-Specific Regulatory Variation Is Related to Sex Bias in Transcript Level

Sex differences in expression were examined using OE, both across genotypes and for each homozygous X^sub^ genotype separately. A majority of genes (84%) showed a significant effect of sex in the overall test. The distribution of sex effects is shown in figure S3, Supplementary Material online. Female-biased expression was more common than male-biased expression (5,380 vs. 3,444 genes, respectively), consistent with previous studies (Ranz et al. 2003; however, see Zhang et al. 2007). There was no evidence for genetic variation for sex bias (supplementary table S6, Supplementary Material online).

Gene location impacts sex bias in *D. melanogaster*: there are reports of fewer male-biased genes on the X chromosome (Parisi et al. 2003; Baines et al. 2008). Similarly, for our set of *D. simulans* genotypes, we find more female-biased genes relative to male-biased genes on the X relative to the autosomes (table 2; χ^2^: *P* < 0.0001). For X-linked genes, sex differences in transcript abundance may result either via the sex determination pathway (e.g., downstream of *fru* and *dsx*; Christiansen et al. 2002) or from dosage compensation (Baker and Ridge 1980; Christiansen et al. 2002; Straub and Becker 2007); while for genes on autosomes, differences in transcript abundance between the sexes are expected to result solely from the sex determination pathway (*trans* variation via sex determination, or *cis* variation in the binding sites for *dsx,* etc.).

**Table 2:** Sex Bias by Chromosomal Location (*n* = 10,422)

Bias Direction/Chromosome	Male Bias	Female Bias	No Bias
X	386 (498)	913 (778)	208 (231)
Autosome	3,058 (2946)	4467 (4,602)	1,390 (1,367)

Note.—The observed (expected) number of genes in each category is shown. There are more female-biased genes and fewer male-biased genes on X than expected (χ^2^: *P* < 0.0001).

For autosomal genes, *cis* and *trans* tests generally showed agreement across the sexes, with ∼82% of tests consistent across sexes (fig. 2, contrasts 1 and 3; supplementary fig. S4*A* and *B*, Supplementary Material online). However, some tests (i.e., *cis* or *trans*) were significant only in females, or only in males; we refer to these as sex-limited (or, female- or male-limited as appropriate), though of course it is always possible that the other sex does have some variation, but so little that we could not detect it. For both *cis* and *trans*, significantly more genes have female-limited variation than male-limited variation (McNemar’s test: *P* < 0.0001). The number of genes with female-limited or male-limited regulatory variation is related to sex bias (table 3), but the nature of relationship is dependent on whether *cis* or *trans* variation is considered. Genes with female-limited, significant *trans* variation tended to be female-biased, while genes with male-limited *trans* variation tended to be male-biased. Overall, sex-biased genes showed more *trans* variability than unbiased genes. Considering *cis* variation, a different association was found: genes that showed female-limited *cis* variation are overrepresented among male-biased genes, while genes with male-limited *cis* variation are slightly overrepresented among female-biased genes.

**Table 3:** Sex-Biased Genes Differ in Sex-Limited Regulatory Variation

Sex-Bias Class	No *Cis*	Male-Only *Cis*	Female-Only *Cis*	Both Sexes *Cis*
*A. Cis*				
No bias	709 (699)	55 (101)	127 (158)	499 (432)
Female bias	2,298 (2,247)	367 (325)	396 (507)	1,405 (1,387)
Male bias	1,477 (1,538)	227 (223)	489 (347)	864 (949)

Sex-Bias Class	No *Trans*	Male-Only *Trans*	Female-Only *Trans*	Both Sexes *Trans*
*B. Trans*				
No bias	1,103 (1,038)	79 (92)	97 (149)	111 (111)
Female bias	3,201 (3,337)	131 (295)	726 (480)	409 (356)
Male bias	2,355 (2,284)	379 (202)	134 (328)	190 (244)

Note.—There are 8,915 autosomal genes for which sex bias (i.e., higher expression in one sex relative to the other) and *cis*/*trans* variation could be compared. The observed (expected) number of genes in each category is given for *cis* (*A*) and *trans* (*B*). *A*. *Cis* variation: Female-limited *cis* variation is overrepresented among male-biased genes and underrepresented among female-biased genes. Male-limited *cis* variation is overrepresented among female-biased genes, although to a lesser degree, than is true for female-limited *cis* variation. Male-limited *cis* variation is underrepresented among unbiased genes. *B*. *Trans* variation: Female-specific regulatory variation is overrepresented among female-biased genes, while male-biased genes are underrepresented. Male-biased genes show the reverse pattern. The number of genes in the male-only and female-only classes significantly differ from one another in all cases (McNemar’s test; *P* < 0.0001), except for female-biased genes in (*A*) and unbiased genes in (*B*). The patterns observed for each class of genes, Female bias, male bias, and no bias also significantly differ from one another (Breslow-Day test for homogeneity of the odds ratios; *P* < 0.0001 for *cis* and *P* < 0.006 for *trans*).

### X-Linked Genes in Females Are Depleted for Both *Cis*- and *Trans*-Regulatory Variation Relative to Autosomal Genes

**Fig. 4.—:**
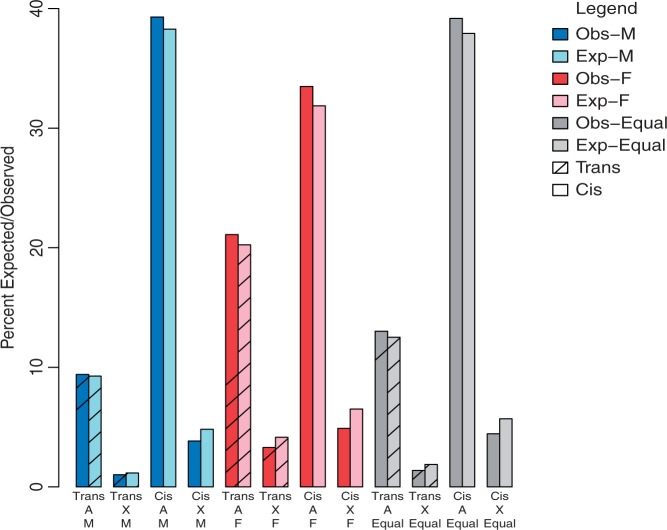
Depletion of regulatory variation on the X relative to autosomes. The percent of genes that vary in *cis* (solid) or *trans* (diagonal shading) regulation (F_1_ test) relative to the percent expected (light bars) for genes on X and genes on autosomes. Results are shown for male-biased (blue), female-biased (red), and equally expressed (gray) genes. For *trans* variation, only female-biased genes had significantly less variation on X than expected (*P* = 0.0001). Whereas depletion of *cis* variation on X is unrelated to sex bias (females, *P* < 0.0001; males, *P* = 0.0031; and unbiased *P* = 0.0002).

Although X-linked genes have a propensity to be female biased and there is more female-specific variation overall, a greater percentage of autosomal genes show *trans* variation than X-linked genes (fig. 4; χ^2^: *P* = 0.001). However, when male-biased, female-biased, and unbiased genes are considered separately, only female-biased genes show significantly less *trans* variation on X, given results from the autosomes (fig. 4; χ^2^: *P* = 0.0001). This may be because the X spends more time in females, and hence selection is more efficient for female-specific *trans* variation affecting X-linked loci. For *cis* variation, the proportion of genes is also significantly different between X and autosomes, again with fewer genes than expected on X (χ^2^: *P* < 0.0001). The depletion of *cis* variation on X was significant even when female (χ^2^: *P* < 0.0001), male (χ^2^: *P* = 0.003), and unbiased (χ^2^: *P* = 0.0002) genes were considered separately; however, the difference is greatest among female-biased genes (fig. 4).

Interestingly, we also observe significantly less *cis* variation in female-biased genes than in male-biased genes on the autosomes (χ^2^: *P* = 0.02). There is a similar trend for the X, but it is not significant (χ^2^: *P* = 0.49). The simplest explanation for this observation is that female-biased genes are under stronger purifying selection than male-biased genes, perhaps due to their association with more severe deleterious phenotypes in both sexes (Connallon and Clark 2011). In contrast, a higher percentage of female-biased genes show *trans* variation relative to male-biased genes; this pattern is significant for both the autosomes (χ^2^: *P* < 0.0001) and for the X (χ^2^: *P* < 0.0001). *Trans* effects are associated with nonadditive sources of variation (Lemos et al. 2008; Gruber et al. 2012), and thus these results are consistent with patterns of regulatory variation found in *D. melanogaster* by Wayne et al. (2007), where variation for transcript abundance in females was found to be more frequently nonadditive than in males.

## Discussion

Here we have used analysis of ASE to identify genes within *D. simulans* with significant *cis* and *trans* variation in five X-substitution heterozygotes, in F_1_ genotypes, and in their respective homozygous parental lines. These experiments allow for a direct comparison of several factors that have previously been considered separately. Our results show that the observation of a greater contribution of *cis*-regulatory variants, relative to *trans* acting variants, to transcript-level variation is a general phenomenon-transcending approach, sex or gene location (X vs. autosomes).

Overall, the number of genes showing evidence of *cis* variation was nearly double the number showing *trans*-acting variation, consistent with previous studies (Lemos et al. 2008; Wittkopp et al. 2008b). Close to 20% of all genes show evidence of *trans* variants. This is unsurprising, as at least some *trans*-acting variation should be caused by nonsynonymous mutations (i.e., protein variants in transcription factors), and approximately 95% of genes in these strains have at least one nonsynonymous mutation (Begun et al. 2007).

Two nonexclusive hypotheses are consistent with greater *cis* than *trans* variation. First*, trans* variation may be relatively more deleterious than *cis* variation, potentially because of extensive pleiotropy (Brem et al. 2002; Yvert et al. 2003; Denver et al. 2005; Prud'homme et al. 2007; Wittkopp et al. 2008b; Gruber et al. 2012). The lack of large *trans* effects in both sexes is consistent with the hypothesis of extensive pleiotropy. By this argument, *trans*-acting variation may be eliminated from the population more frequently than *cis*-acting variation, thus explaining the relative abundance of *cis* variation within species (Lemos et al. 2008; Wittkopp et al. 2008b; Gruber et al. 2012).

The second hypothesis explaining greater *cis* than *trans* variation is that we observe a smaller fraction of the extant genetic variation for *trans* than for *cis*, due to summing of effects across *trans* mutations for a given focal gene, rather than measuring the individual effects of each *trans* mutation. Combining small effect variants of opposite sign may result in a sum of close to zero (Barton and Turelli 1989; Griswold and Whitlock 2003), resulting in an apparently smaller contribution of *trans* variance. Alleles whose effects cancel each other out could also result in an overall smaller range (as distinct from a smaller mean) in the size of significant *trans* estimates, as we observe here.

We might expect to see such an underestimate for *trans*, but not for *cis*, for a variety of biological reasons. First, the mutational target size for *trans* variation might well be larger than that for *cis* variants, because *trans*-acting factors are themselves the products of both *cis* and *trans* variation, and because multiple *trans*-acting factors may act on a single focal gene (possibly via long regulatory cascades). Moreover, nonadditivity, which is more common for *trans* than for *cis* variation (Lemos et al. 2008; Gruber et al. 2012), can contribute to longer transit times in the population (Kimura and Ohta 1969). 

How does sex affect *cis-* and *trans*-regulatory variability? Under sex-specific selection, we expect that more variation will be observed in the sex for which fitness consequences of regulatory variation are less important. If we accept that sex-biased expression is an indication of sex-specific function, we can test this hypothesis. We found that there is more male-specific *cis*-regulatory variation among female-biased genes than there is female-limited *cis* variation; and conversely, that there is more female-specific than male-limited *cis* regulatory variation among male-biased genes. These results are consistent with decoupling of fitness effects between sexes for *cis*-acting mutations. Female-biased genes have less than expected levels of female-limited *cis* variation (table 3), consistent with stronger or more efficient selection in females. Male-biased genes show close to random levels of male-limited variability.

*Trans* variation is fundamentally different from *cis* variation with respect to sex bias. Male-biased genes showed less female-limited variation, while female-biased genes have less male-limited variation. And for autosomal genes, female-biased genes actually had greater than expected levels of female-limited *trans* variation, while male-biased genes showed an opposite pattern. One possible explanation for this pattern is sex-limited expression of the genes coding for the *trans*-acting factors that regulate sex-biased genes. This would result in mutations that can only affect expression in one sex. *Cis*-regulatory variants, in contrast, are less frequently sex-limited and thus would generally be expected to affect expression in both sexes, except in cases of *cis* × *trans* interactions, which could similarly cause sex-limited effects.

There is a fundamental symmetry between the sexes with regard to the relationship between sex bias and sex-limited variation. Males and females show similar patterns of sex-limited *cis* and *trans* variation, even though the pattern shared by the sexes for *trans* is different from the pattern they share for *cis*.

Despite overall similarities between the sexes with respect to the pattern of regulatory variation, there remain stark differences between the X and the autosomes. Male hemizygosity combined with recessivity of factors on the X should result in greater efficiency of selection on the X than on the autosomes for genes that are functionally relevant in males (Charlesworth et al. 1987). This process should result in reduced variability on X relative to autosomes (Gordo and Charlesworth 2001; Vicoso and Charlesworth 2006). To the extent that sex bias indicates function in the biased sex (Connallon and Clark 2011), purifying selection should be more efficient for male-biased genes than for female-biased genes, given partial recessivity.

Given recessivity of a portion of *cis* variation, theory clearly predicts that *cis* variation should be depleted on X as the underlying causal variants are X-linked. This should be especially apparent in male-biased genes. We found that *cis* variation was depleted on X for male-, female- and unbiased genes. However, the greatest depletion was observed among female-biased genes, as opposed to male-biased genes. Our results are consistent with other studies demonstrating lower variation on the X for expression either overall (Lawniczak et al. 2008) or, in contrast to our results, for male-biased genes only (Llopart 2012). Others have also demonstrated a faster-X effect for gene expression (Baines et al. 2008; Meisel et al. 2012; Meisel and Connallon 2013), which could potentially result in lower within-species variation if selective sweeps are frequent.

*Trans* variants are more likely to harbor dominance variance than *cis* variants (Lemos et al. 2008; Gruber et al. 2012). Moreover, *trans* variation may be more deleterious than *cis* due to greater pleiotropy (Brem et al. 2002; Denver et al. 2005; Prud'homme et al. 2007; Gruber et al. 2012) and hence is expected to be under relatively stringent purifying selection. Assuming some portion of the causal variants are X-linked, we can test the hypothesis of greater efficiency of selection for male-relevant alleles by examining *trans*-acting variation in male-biased genes on the X. There are three important caveats to this test. First, even though the genes whose expression we quantified are on the X, we have no way of knowing whether the genes responsible for the *trans* variation are also X-linked. Second, it is possible that these particular *trans*-acting variants do not meet the recessivity requirement, as they were not significant for dominance variance. Finally, it is possible that dominance for transcript abundance does not translate literally into dominance for fitness (Fry 2010). Accordingly, though we found no evidence of reduced *trans* variation in the 386 male-biased genes that we detected on the X, this result must be considered with caution. However, consistent with this result, it is becoming increasingly clear that sexually antagonistic alleles, though charismatic, are relatively rare (Innocenti and Morrow 2010; Sharp and Agrawal 2013), even on the X (Mallet et al. 2011). 

Surprisingly, there is significant underrepresentation of *trans* variation for female-biased genes on the X relative to the autosomes (*P* = 0.0001). One possible explanation for underrepresentation of *trans* variation on the X for female-biased, but not male-biased, genes is that X spends a disproportionate amount of time in females relative to males (2/3 vs. 1/3). Only sexually antagonistic alleles with recessive, female-negative effects can accumulate on the X via hemizygous advantage. Otherwise, selection should be extremely efficient with respect to female-affecting alleles on the X relative to the autosomes. The enrichment of X for female-biased genes, then, may well be a straightforward outcome of this disproportionate time-sharing arrangement (Meisel et al. 2012). In fact, *trans*-acting factors for female-biased genes are more likely to be encoded on the X than male-biased genes, consistent with arguments for the feminization of the X. We suggest that despite the theoretical arguments about the importance of the X to male-driven evolution and sexual antagonism, the depletion of regulatory variability in X-linked genes is mainly the result of a female-dominated selection regime, and that greater consideration of the evolutionary processes governing evolution of female-biased and female-specific genes is warranted (Lawniczak et al. 2008). Our results point to a need for clear theoretical predictions with respect to the depletion of regulatory variation on X, as well as for additional data. Ideally, such a framework would include consideration of X-linkage of causal variants, the contribution of *cis* by *trans* interactions, sex-biased expression, breadth of expression/tissue-specificity, and sex-specific fitness effects.

## Supplementary Material

Supplementary figures S1–S4, tables S1–S6, and files S1–S2 are available at *Genome Biology and Evolution* online (http://www.gbe.oxfordjournals.org/).

Supplementary Data
