# Supplementary material for: What the X Has to Do with It: Differences in Regulatory Variability between the Sexes in Drosophila simulans
Source: Genome Biol Evol. 2014 Apr 1;6(4):818–29. doi: 10.1093/gbe/evu060 (PMC4007535; doi:10.1093/gbe/evu060)
Supplement: Supplementary Data [file supp_6_4_818__index.html]

What the X has to do with it: differences in regulatory variability between the sexes in Drosophila simulans. — What the X Has to Do with It: Differences in Regulatory Variability between the Sexes in Drosophila simulans — Supplementary Data 

# What the X Has to Do with It: Differences in Regulatory Variability between the Sexes in *Drosophila simulans*

## Supplementary Data

files

**Files in this Data Supplement:**

- Supplementary Data - zip file
