# Supplementary material for: What the X Has to Do with It: Differences in Regulatory Variability between the Sexes in Drosophila simulans
Source: Genome Biol Evol. 2014 Apr 1;6(4):818–29. doi: 10.1093/gbe/evu060 (PMC4007535; doi:10.1093/gbe/evu060)
Supplement: Supplementary Data [file supp_evu060_suppl_data.zip › combined_supplement_revised.pdf]

Table S1a-b. Genotype variability in *cis* and *trans* effects in F_1 female experiments. For each genotype individually, the number of genes with significant *cis* (contrast 1 in Figure 2) and/or *trans* (contrast 3 in Figure 2) variation in the female F_1 tests is reported, comparing X-linked (n = 550 tests) and autosomal (n = 4,276 *cis* tests and n = 4284 *trans* tests) genes. Only results for genes with informative probe sets in all genotypes are reported.

a) X-linked genes

X^{sub} parent	***Cis***		***Trans***	
	**# sig.**	**% sig.**	**Num. sig.**	**% sig.**
c167	163	29.64	103	18.73
md106	28	5.09	10	1.82
md199	11	2.00	12	2.18
Newc	25	4.55	2	0.36
w501	30	5.45	8	1.45

b) Autosomal genes

X^{sub} parent	***Cis***		***Trans***	
	**# sig.**	**% sig.**	**# sig.**	**% sig.**
c167	1322	30.92	878	20.49
md106	383	8.96	55	1.28
md199	245	5.73	85	1.98
newc	336	7.86	30	0.70
w501	191	4.47	40	0.93

Table S2. Genotype variability in *cis* and *trans* effects in X^{het} *st e* **female experiments.** For each genotype individually, the number of genes with significant cis and/or trans variation in the female X^{het} *st e* tests is reported, comparing *cis* (contrast 2 in Figure 2) and *trans* (contrast 4 and 5 in Figure 2). Only results for genes with informative probe sets in all genotypes are reported (n = 550).

X^{sub} parent	*Cis*		*Trans (4)*		*Trans(5)*	
	**# sig.**	**% sig.**	**# sig.**	**% sig.**	**# sig.**	**% sig.**
C167.4	99	18.00	4	0.73	30	5.45
Md106	15	2.73	0	0.00	3	0.55
Md199	14	2.55	2	0.36	12	2.18
NewC	19	3.45	1	0.18	0	0.00
W501	26	4.73	2	0.36	1	0.18

Table S3. Genotype variability in *cis* and *trans* effects in F_1 male experiments. For each genotype individually, the number of genes with significant cis (n = 4276 tests) and/or trans (n = 4284 tests) variation in the male F_1 tests is reported, for autosomal genes. Only results for genes with informative probe sets in all genotypes are reported.

**X^{sub} parent**	***Cis***		***Trans***	
	**# sig.**	**% sig.**	**# sig.**	**% sig.**
C167.4	1047	24.49	570	13.31
Md106	360	8.42	35	0.82
Md199	242	5.66	103	2.40
NewC	333	7.79	37	0.86
W501	190	4.44	47	1.10

Table S4. Genes with *cis* by *trans* interaction variation. For each of the X^{sub} parental genotypes the F_1 and X^{het} *st e* can be compared for genes on the X. Differences in *cis* effects between the F_1 ($X^{sub}X^{st\ e}A^{sub}A^{st\ e}$) and the X^{het} *st e* ($X^{sub}X^{st\ e}$,$A^{st\ e}A^{st\ e}$) mean that a *cis* effect is dependent on *trans* acting factors in the background. This indicates a role for *cis* by *trans* interactions in the regulatory differences observed. Only results for genes with informative probe sets in all genotypes are reported (n=550).

X^{sub} parent	# Sig.	% Sig.
c167.4	26	4.73
md106	7	1.27
md199	8	1.45
newc	0	0
w501	0	0

Table S5. Summary statistics. For each test type, indicated by the parental X-sub genotype corresponding to the genotypes examined in the test, summary statistics are reported for the raw estimate of the effect corresponding to the test listed. The Chr. Column indicates that the estimates are for X-linked or for autosomal genes. The directionality of the estimates are summarized by the column # pos. est. and # neg. est., which list the number of positive and negative estimates respectively.

X^{sub} parent	Test	Sex	Chr.	*N*	Median	Mean	σ	# pos. est.	# neg. est.
C167.4	trans, contrast 3	Female	A	1490	-0.5406	-0.5328	0.2661	38	1452
Md106	trans, contrast 3	Female	A	147	-0.3831	-0.1097	0.6167	46	101
Md199	trans, contrast 3	Female	A	209	-0.4447	-0.2233	0.5403	44	165
NewC	trans, contrast 3	Female	A	79	-0.4304	-0.08851	0.6216	25	54
W501	trans, contrast 3	Female	A	72	-0.4675	-0.25	0.5457	13	59
C167.4	trans, contrast 3	Female	X	211	-0.5454	-0.5476	0.2313	3	208
Md106	trans, contrast 3	Female	X	22	-0.46	-0.3401	0.6495	5	17
Md199	trans, contrast 3	Female	X	27	-0.4523	-0.3048	0.5839	4	23
NewC	trans, contrast 3	Female	X	11	-0.5219	-0.1907	0.6298	3	8
W501	trans, contrast 3	Female	X	12	0.5565	0.1593	0.6541	7	5
C167.4	trans, contrast 4	Female	X	10	0.4061	0.4894	0.2451	10	0
Md106	trans, contrast 4	Female	X	5	0.8499	1.004	0.49	5	0
Md199	trans, contrast 4	Female	X	6	0.4936	0.7177	0.4144	6	0
NewC	trans, contrast 4	Female	X	4	0.5664	0.3007	0.6377	3	1
W501	trans, contrast 4	Female	X	2	0.05152	0.05152	0.6915	1	1
C167.4	trans, contrast 5	Female	X	62	0.6308	0.3876	0.6997	45	17
Md106	trans, contrast 5	Female	X	17	-0.8056	-0.4398	0.7879	5	12
Md199	trans, contrast 5	Female	X	47	0.7538	0.5946	0.7262	38	9
NewC	trans, contrast 5	Female	X	5	-0.5253	0.01012	0.8226	2	3
W501	trans, contrast 5	Female	X	2	-0.6176	-0.6176	0.0459	0	2
C167.4	trans, contrast 3	Male	A	1096	-0.4838	-0.5226	0.2941	21	1075
Md106	trans, contrast 3	Male	A	91	-0.4653	-0.3723	0.5065	14	77
Md199	trans, contrast 3	Male	A	212	-0.4785	-0.3625	0.4385	28	184
NewC	trans, contrast 3	Male	A	87	-0.4797	-0.4556	0.3747	5	82
W501	trans, contrast 3	Male	A	84	-0.4887	-0.3585	0.4765	10	74
C167.4	cis, contrast 1	Female	A	2655	-0.4625	-0.2046	0.6318	817	1838
Md106	cis, contrast 1	Female	A	1160	-0.5068	-0.4742	0.3777	107	1053
Md199	cis, contrast 1	Female	A	730	-0.6049	-0.5175	0.4995	117	613
NewC	cis, contrast 1	Female	A	1023	-0.604	-0.5637	0.4073	86	937
W501	cis, contrast 1	Female	A	327	-0.621	-0.5135	0.5918	62	265
C167.4	cis, contrast 1	Female	X	369	-0.3465	0.01743	0.6917	172	197
Md106	cis, contrast 1	Female	X	91	-0.6038	-0.4786	0.4648	12	79
Md199	cis, contrast 1	Female	X	40	-0.6934	-0.489	0.6803	9	31
NewC	cis, contrast 1	Female	X	89	-0.6335	-0.6072	0.4149	5	84
W501	cis, contrast 1	Female	X	53	-0.7994	-0.6888	0.4856	5	48
C167.4	cis, contrast 2	Female	X	246	-0.4967	-0.2922	0.589	62	184
Md106	cis, contrast 2	Female	X	50	-0.6208	-0.4156	0.6498	9	41
Md199	cis, contrast 2	Female	X	49	0.4051	-0.01545	0.8539	25	24
NewC	cis, contrast 2	Female	X	67	-0.6665	-0.6382	0.4254	4	63
W501	cis, contrast 2	Female	X	44	-0.8328	-0.8305	0.2946	1	43
C167.4	cis by trans, contrast 6	Female	X	57	0.3795	0.1857	0.4483	45	12
Md106	cis by trans, contrast 6	Female	X	31	-0.4297	-0.3381	0.5686	6	25
Md199	cis by trans, contrast 6	Female	X	17	-0.5572	-0.6875	0.2593	0	17
C167.4	cis, contrast 1	Male	A	2114	-0.406	-0.1435	0.6251	727	1387

Md106	cis, contrast 1	Male	A	1087	-0.497	-0.4569	0.3511	94	993
Md199	cis, contrast 1	Male	A	720	-0.5777	-0.4975	0.4738	111	609
NewC	cis, contrast 1	Male	A	993	-0.5645	-0.5341	0.3759	80	913
W501	cis, contrast 1	Male	A	343	-0.6236	-0.5097	0.5616	63	280

Table S6. Test for sex differences in individual genotypes. For 12,931 genes (19,339 probesets) we were able to test for a difference in expression between the sexes for overall transcript level using the Affymetrix probesets for 3' IVT expression from the Drosophila 2.0 array.

X^{sub} parent	Male Biased[a]	Female Biased[a]
C167.4	3,273 (4,814)	4,087 (7,171)
Md106	3,174 (4,820)	4,276 (7,165)
Md199	3,337 (4,775)	4,210 (7,210)
NewC	3,197 (4,757)	4,897 (7,228)
W501	3,266 (4,731)	4,497 (7,254)

Figure S1. DNA controls for allele specific analysis of *cis* and *trans* regulatory variation. To account for technical limitations, the expected relationships between allele-specific hybridization signals were estimated directly from DNA control hybridizations. The allele specific DNA hybridization signal in the F_1 was used as a control for *cis* tests, in which the null expectation is equal expression. Allele-specific DNA hybridization signals in parental strains and in the F_1 genotypes are used as controls for the *trans* tests, in which the null expectation is that parental expression is twice the allele specific expression in the F_1. For each contrast 1-6: the genotypes (only c167.4 shown) used as DNA controls are shown in the first column; for a given focal gene, the allele specific signals used in the test are given in the second column, noted as C (allele derived from the C167.4 parental strain) or S (allele derived from the *st e* reference strain) in the genotype indicated by subscripts P (parental strain), F1 (F_1), or X1 (X^{het} *st e*); the genes that could be tested, X-linked (X) and autosomal (Autosome), are listed in the third column; the sex, Male (M) and Female (F), that the test could be conducted for is listed in the fourth column (with the genes that could be tested in superscript); and the effect tested is listed in the fifth column.

Figure S2A-C. *Cis*, *trans* and *cis* by *trans* variation in transcript abundance. Variation in transcript abundance resulting from variation in *cis* regulatory regions of a focal gene (contrast 1 only), in *trans* acting factors regulating the gene (contrast 3 only), or resulting from genetic interactions between them (contrast 6), were cross-compared for X-linked genes in females (a). For autosomal genes, the overlap of *cis* (contrast 1 only) and *trans* (contrast 3 only) based variation in transcript abundance is shown in females (b) and in males (c). The area representing genes with *cis* only variation is shaded in blue, the area representing genes with *trans* only variation is shaded in pink and the area representing genes with only *cis* by *trans* variation is shaded in yellow. The number of genes that were tested for each group, but were not significant, is shown next to the diagram.

Figure S3. The distribution of sex differences in transcript abundance. The Y-axis is the count and the X-axis is the estimate of the sex effect from the overall test of sex differences in transcript abundance. Negative values are biased toward greater male expression and positive values toward greater female expression.

Figure S4A-B. Sex specific *cis* and *trans* regulatory variation. A Venn diagram showing the overlap among autosomal genes with significant variation (FDR < 0.20) in males (pink) and females (blue) for (A) *cis* (contrast 1 only) and (B) *trans* (contrast 3 only) is shown. The number of genes that were tested for *cis* or for *trans* variation in transcript abundance in both sexes, but were not significant, is shown next to the diagram.

Genotypes Used in Contrast	Alleles Compared in Contrast	Chr. Tested	Sex$^{chr.}$	Type
1) F_1 — X, 2, 3	1) $S_{DNA\text{-}F1}$ - $C_{DNA\text{-}F1}$	X, autosomes	$F^{X,A}$	*cis*
2) X^{het} *st e*	2) $S_{DNA\text{-}X1}$ - $C_{DNA\text{-}X1}$	X	F^{X}	*cis*
3) Homozygous *st e*; F_1 — X, 2, 3	3) $S_{DNA\text{-}P}$ - $S_{DNA\text{-}F1}$	X, autosomes	$F^{X,A}$	*trans*
4) Homozygous *st e*; X^{het} *st e*	4) $S_{DNA\text{-}P}$ - $S_{DNA\text{-}X1}$	X	F^{X}	*trans*
5) X^{sub}; X^{het} *st e*	5) $C_{DNA\text{-}P}$ - $C_{DNA\text{-}X1}$	X	F^{X}	*trans*

Allele-specific DNA hybridization signal

Female F

X-substitution strain

st e strain

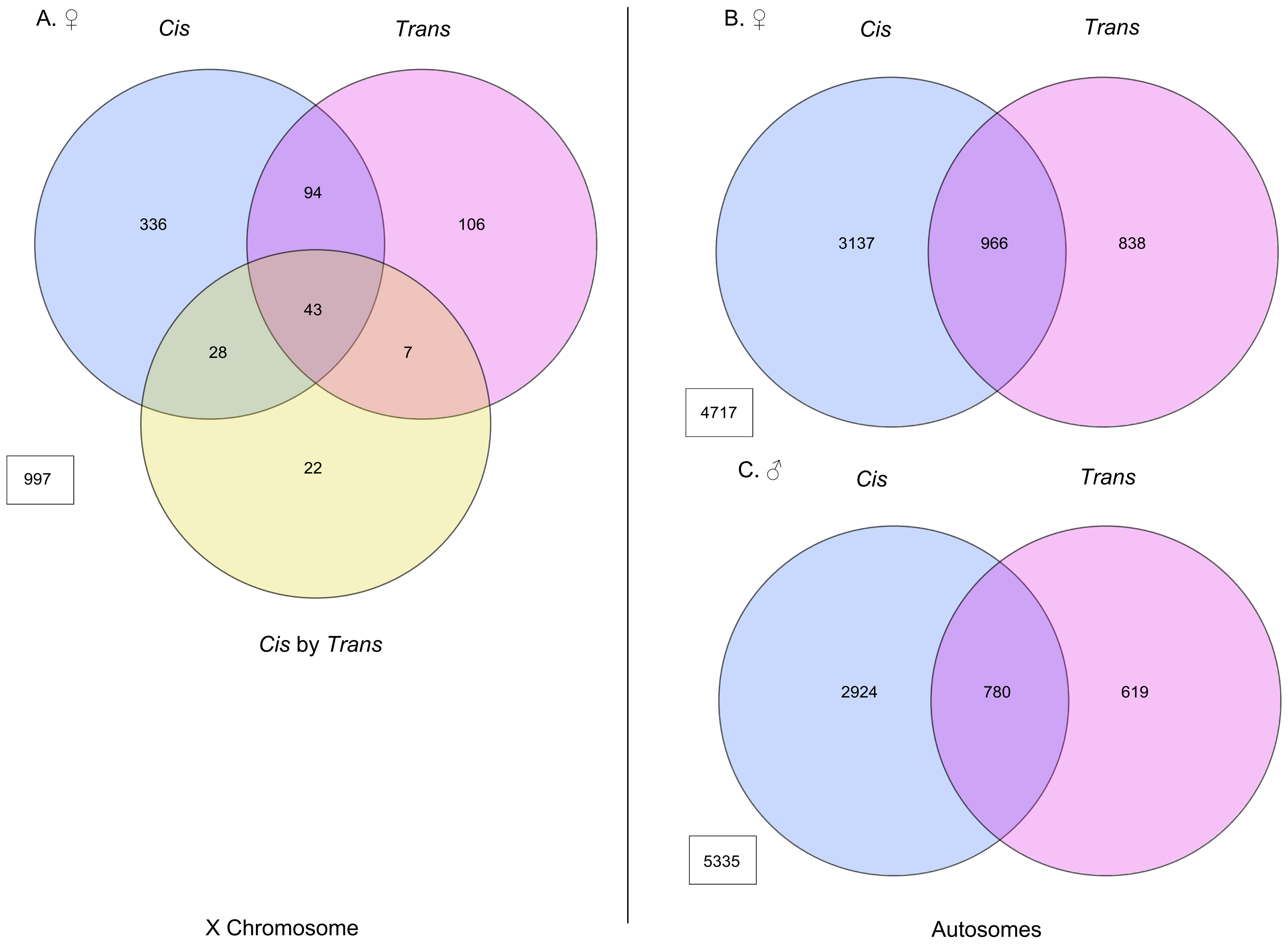
A. ♀
Cis
Trans
336
94
106
43
28
7
22
997
Cis by Trans
X Chromosome
B. ♀
Cis
Trans
3137
966
838
4717
C. ♂
Cis
Trans
2924
780
619
5335
Autosomes

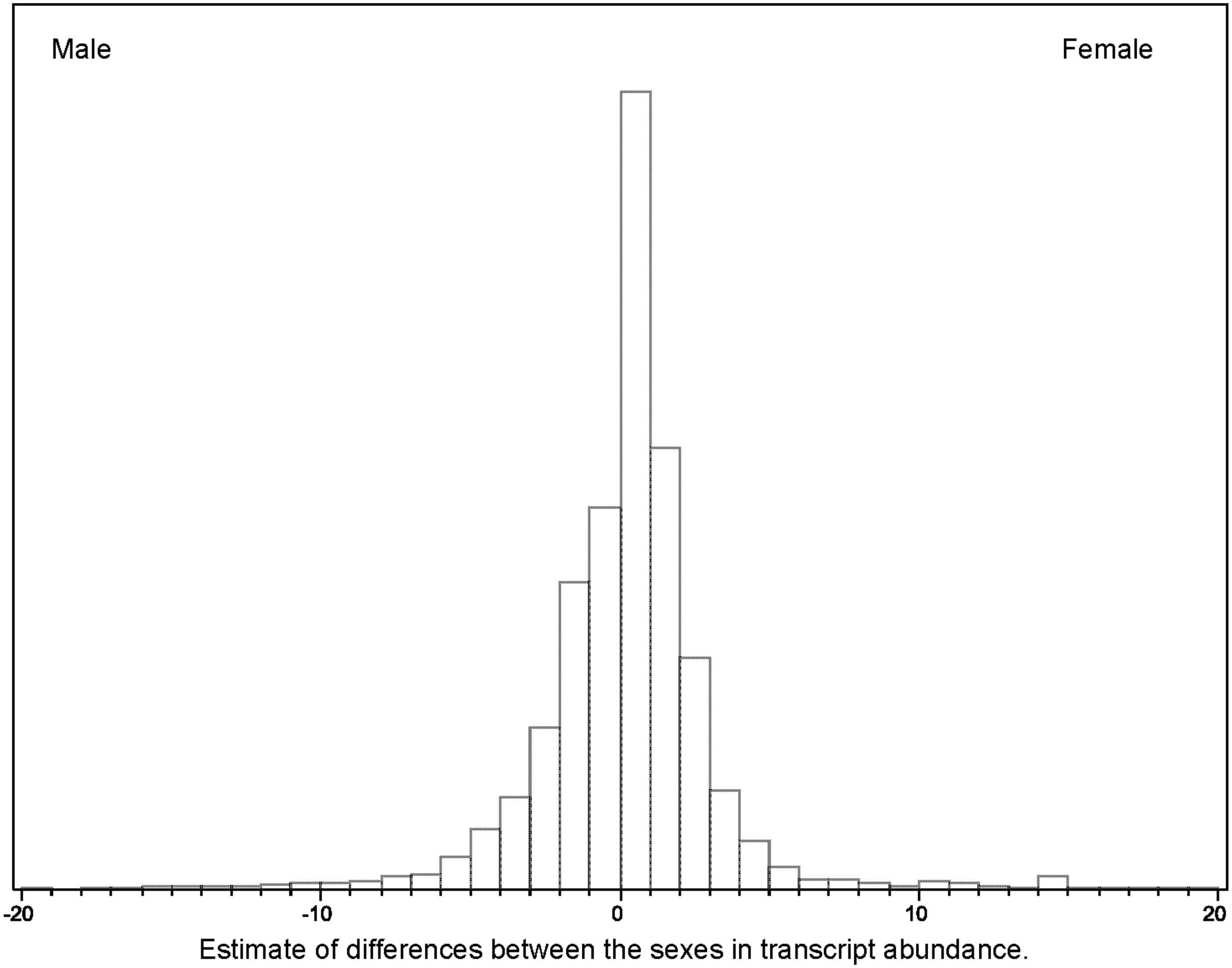
Male
Female
-20
-10
0
10
20
Estimate of differences between the sexes in transcript abundance.

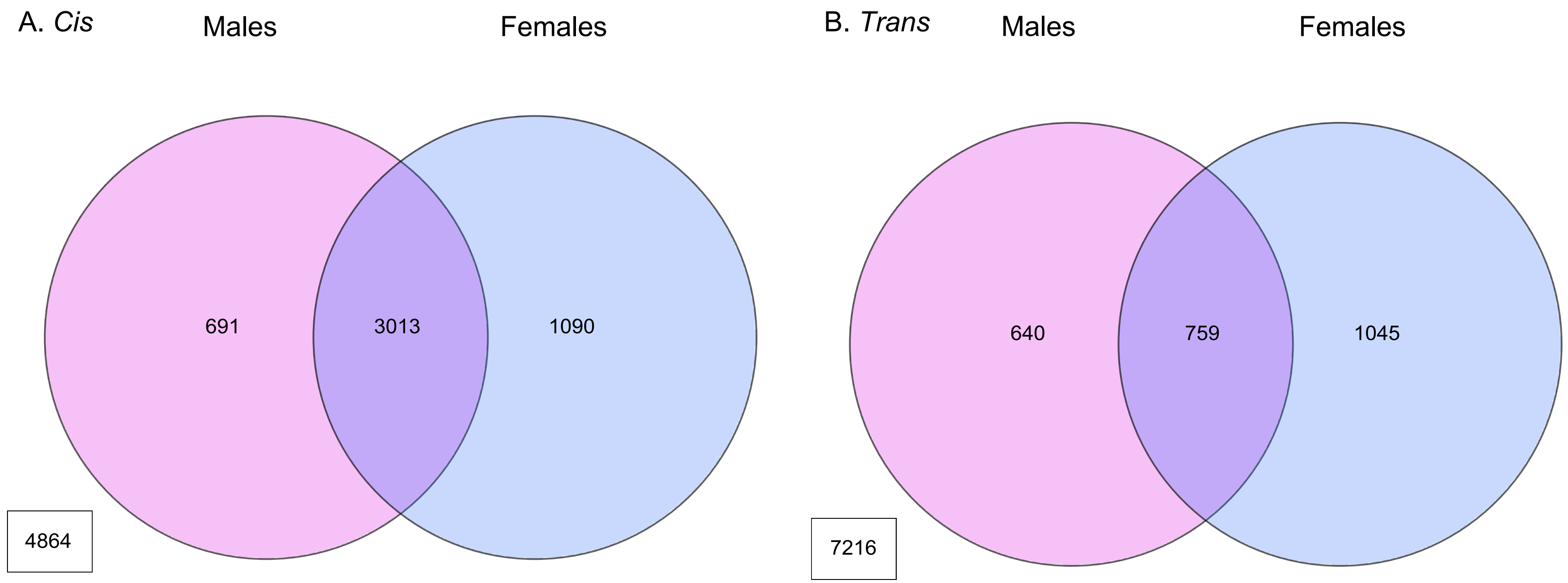
A. Cis
Males
Females
691
3013
1090
4864
B. Trans
Males
Females
640
759
1045
7216
